# Force–Vision Multimodal Measurement of Model-Equivalent Elastic Parameters for Soft Tissue Modeling

**DOI:** 10.3390/biomimetics11100691

**Published:** 2026-09-24

**Authors:** Le Zhang, Yi Liu, Ziyu Liang, Dapeng Yang

**Affiliations:** State Key Laboratory of Robotics and Systems, Harbin Institute of Technology, Harbin 150080, China; 21b908034@stu.hit.edu.cn (L.Z.); 24s008075@stu.hit.edu.cn (Y.L.); 25s108164@stu.hit.edu.cn (Z.L.)

**Keywords:** soft tissue modeling, model-equivalent parameter identification, force–vision sensing, finite-element contact modeling, Bayesian optimization

## Abstract

In robotic manipulation, robot-assisted surgery, and other contact-rich interactions with deformable bodies, reliable prediction of contact force and deformation requires rapid determination of the effective mechanical parameters governing the soft-tissue model. However, these model-equivalent properties are difficult to obtain directly from physical interaction, particularly for human soft tissue with substantial variability in geometry and mechanical response. This work presents a force–vision multimodal framework for rapidly identifying effective elastic and contact-model parameters from measured force and deformation. The human breast is selected as a representative validation object because of its high compliance, pronounced geometric variability, and sensitivity of its deformation response to mechanical properties and contact conditions. A six-axis force/torque sensor and an eye-in-hand RGB-D camera provide time-synchronized measurements of three-dimensional contact force and surface-marker displacement. A finite-element contact model maps the probe pose and a parameter vector to the measured responses, while a multiobjective Bayesian optimization procedure identifies the parameter set under a limited simulation budget. Experiments using three breast-tissue-mimicking phantoms with different geometries and stiffnesses yield normalized force and displacement residuals of 4.93%–6.00% and 7.18%–12.33%, respectively, within 55 model evaluations. Force and displacement provide complementary constraints, and the Bayesian solver achieves residuals comparable to particle swarm optimization with a 5.5-fold reduction in the number of finite-element evaluations. Held-out indentation and controlled posture-change tests further support the predictive consistency of the identified model-equivalent parameters within the experimental protocol. The proposed framework provides an approach for rapid parameter identification of deformable-tissue contact models, with potential to support more consistent reproduction of contact force and deformation in soft-tissue simulation and future sim2real robotic manipulation with force–vision feedback.

## 1. Introduction

Soft-tissue simulation is increasingly used for contact-rich manipulation, deformable-object handling, surgical assistance, and digital-twin construction. Its usefulness depends on whether the simulated object reproduces both the force exchanged at contact and the deformation observed in the physical object. Constraint-based contact formulations and interactive soft-tissue simulators provide the numerical foundation for this class of problems [1,2]. Recent studies on compliant manipulation and deformable-object interaction further show that geometric uncertainty, object compliance, contact history, and changing boundary conditions can strongly affect planning and control [3,4,5,6]. A simulator that uses arbitrary or poorly calibrated parameters can therefore produce visually plausible deformation while still predicting incorrect contact forces, grasp stability, or interaction trajectories.

The central difficulty is that the parameters required by a robot simulator are not necessarily identical to directly measurable material constants. A soft tissue may be heterogeneous, nonlinear, viscoelastic, multilayered, or strongly dependent on its geometry and boundary constraints. For example, nonlinear finite-element formulations of growing skin and soft biological tissue incorporate hyperelasticity, large deformation, and compressible or nearly incompressible behavior, illustrating the richer constitutive descriptions needed for detailed tissue mechanics [7,8]. Object-specific and patient-specific biomechanical pipelines therefore commonly combine imaging-derived geometry with constitutive assumptions and boundary-condition models [9,10,11]. Breast-shaped and tissue-mimicking bodies provide useful controlled examples because their deformation varies substantially with loading direction, posture, and compression level [12,13]. For simulation and sim2real transfer, the practically useful quantity is therefore often a model-equivalent parameter set: parameters conditioned on the selected geometry, constitutive approximation, boundary conditions, and contact formulation such that the forward model reproduces the measured physical response. This interpretation avoids treating effective parameters fitted from sparse observations as unique ground-truth material constants.

Comparable model dependence also arises in architected metamaterials. Zarastvand et al. used analytical and finite-element models to relate topology-dependent effective mechanical properties of triply periodic minimal-surface (TPMS) sandwich cylinders to frequency-dependent wave propagation and sound-transmission loss [14]. Whereas that work predicts dynamic vibroacoustic response in designed periodic structures, the present study solves a quasi-static inverse problem for compliant phantoms. Both settings require effective quantities to be interpreted together with geometry, loading regime, boundary conditions, and model assumptions.

Obtaining such a parameter set is an inverse problem. In inverse finite-element or model-updating approaches, candidate parameters are repeatedly passed through a forward solver and adjusted to reduce discrepancies between simulated and measured responses. Indentation-based approaches have been applied to soft-tissue constitutive identification, robot-assisted force-based characterization, and finite-element model updating [15,16,17,18]. These studies also expose several practical limitations: force-only measurements may not uniquely constrain multiple parameters, dense displacement fields may be unavailable during physical contact, and repeated nonlinear contact simulations can be computationally expensive. Bayesian optimization and surrogate-assisted strategies have consequently attracted attention in computational mechanics and inverse finite-element problems [19,20,21], while particle-swarm and gradient-based least-squares methods remain useful comparison baselines [22]. The relevant question is therefore not merely which optimizer minimizes a scalar objective but whether the available measurements provide complementary physical constraints and whether the resulting calibrated model remains predictive beyond the observations used for fitting.

The mechanical response under contact cannot be completely observed from a single sensing modality. A force/torque sensor measures interaction load and contact onset but does not determine the spatial deformation of the object. Conversely, optical sensing provides surface geometry or marker displacement but does not directly reveal the corresponding mechanical reaction. RGB-D reconstruction, stereo estimation, point-cloud registration, and surface reconstruction have made non-contact geometry acquisition increasingly practical [23,24], yet geometry alone remains insufficient for predicting contact forces. Combining synchronized force and vision measurements therefore transforms parameter calibration into a multimodal system-identification problem: the force channel constrains the global reaction and effective stiffness level, whereas the vision channel constrains the spatial pattern and magnitude of deformation. A parameter set fitted using only one modality may consequently remain mechanically or geometrically inconsistent with the omitted observations [15,16,17].

This multimodal viewpoint is also closely related to robotic perception of compliant objects. During human interaction with a deformable object, visual observation and contact feedback are naturally integrated during exploratory touch rather than relying on prior knowledge of a unique material modulus. Robotic ultrasound and other probe-based systems provide a representative example of repeatable soft-tissue interaction in which force regulation, surface reconstruction, motion compensation, and sensing feedback must be coordinated [25,26,27,28,29,30,31,32]. In the present study, however, the ultrasound probe serves primarily as a repeatable contact tool, while the breast-shaped phantom provides a controlled compliant object for model identification. Accordingly, this work investigates the calibration of a physics-based soft-tissue representation from synchronized force–vision observations, with the aim of reproducing both contact mechanics and observable deformation for subsequent simulation and contact-aware robotic interaction.

The contributions of this work are fourfold: (1) a synchronized force–vision sensing framework that captures three-dimensional contact force together with sparse surface deformation during controlled soft-tissue interaction; (2) a finite-element contact model that links the recorded probe motion and model-equivalent parameters to both force and deformation observations; (3) a sample-efficient multiobjective Bayesian identification strategy that jointly exploits the complementary mechanical and geometric constraints provided by the two sensing modalities; (4) systematic phantom experiments evaluating residual balance, computational efficiency, modality complementarity, held-out prediction, and deformation under changed boundary conditions. Rather than seeking unique intrinsic material constants, the proposed framework identifies a model-equivalent soft-tissue representation conditioned on the adopted geometry, constitutive model, boundary conditions, and contact formulation. Such a representation provides a physics-consistent interface between multimodal physical interaction and simulation, with potential relevance to active compliant-object perception, contact-aware grasp planning, and sim2real robotic manipulation.

## 2. Materials and Methods

### 2.1. Force–Vision Measurement System

As shown in Figure 1, the sensing system combines a repeatable instrumented probe motion with contact-force sensing and RGB-D surface measurement. The system consists of a robotic manipulator (UR5, Universal Robots, Odense, Denmark), an ultrasound probe used as the controlled contact tool, a six-axis force/torque sensor (KWR75A, Kunwei Technology, Beijing, China), and two RGB-D cameras (Intel RealSense D435i and D405, Intel Corporation, Santa Clara, CA, USA). Camera 1 (D435i) provides coarse phantom localization, whereas Camera 2 (D405) is mounted in an eye-in-hand configuration for high-resolution surface acquisition. The ultrasound image stream is not used as a separate objective in the parameter identification; the probe is used to establish a repeatable soft-object contact geometry. All measurement processing and model evaluation routines are implemented in Python 3.8 on a laptop equipped with an Intel Core i9-13900HX CPU (2.2 GHz) and 16 GB RAM.

### 2.2. Geometry Input to the Measurement Model

As summarized in Figure 2, this geometry is acquired once per phantom by multi-view RGB-D scanning and is thereafter held fixed as an input to the measurement model; it is not an evaluated parameter in this work. The breast region is extracted from the RGB frames by an off-the-shelf instance-segmentation network (YOLOv8m-seg [33]) trained on 400 annotated multi-view images, split 9:1 into 360 training and 40 validation images, and the resulting masks are applied to the corresponding depth frames to suppress background clutter and boundary artifacts. Camera 1 provides a coarse localization of the phantom in the hand–eye calibrated robot base frame, from which Camera 2 acquires 50 approximately uniformly distributed views along a spherical trajectory of radius $R_{1}=2R_{2}$, where $R_{2}$ denotes the estimated breast half-width. Depth is estimated from the stereo pair using FoundationStereo [24] rather than the raw D405 depth, which improves consistency at close range. The views are fused by truncated signed distance function (TSDF) integration with calibrated intrinsics and registered camera poses [34], and the resulting point cloud is converted into a watertight tetrahedral mesh through point-cloud separation, boundary-plane estimation, voxel downsampling, base-plane filtering, Ball-Pivoting surface reconstruction, PyMeshFix repair, and automatic meshing with Gmsh.

### 2.3. Sensed Quantities, Measurement Model, and Inverse Evaluation

#### 2.3.1. Sensed Quantities: Contact Force and Surface Displacement

As shown in Figure 1 and Figure 3a, the two quantities acquired by the sensing system are the probe–phantom contact force and the phantom surface deformation quantified by marker displacements. The contact force is measured in real time using a calibrated six-axis force/torque (F/T) sensor mounted between the robot end-effector and the ultrasound probe, while surface deformation is obtained via marker-based 3D displacement measurement from RGB-D data. Specifically, eight red circular markers are attached to the phantom surface to serve as reference points. Following the geometry measurement procedure in Section 2.2, Camera 2 captures RGB-D frames of the phantom surface, and the corresponding point cloud is transformed from the camera frame to the robot base frame using the hand–eye calibration. The markers are then segmented in the RGB image using HSV thresholding, and their associated 3D points are clustered via K-means ($k_{num}=8$) to estimate the 3D coordinates of the eight markers. Finally, the marker indices are assigned deterministically based on their projected locations on the XY plane (sorted by ascending *Y* and then ascending *X*), as illustrated in Figure 3a, which ensures consistent correspondence of marker measurements across repeated trials. The eight markers provide sparse local observations of surface motion rather than a full-field deformation measurement. Measurement uncertainty arises from RGB-D reconstruction, marker localization, and coordinate transformation and may have a greater relative influence when the observed motion is small. These contributions were not independently quantified in the present experiments.

#### 2.3.2. Measurement Model of the Sensing System

The two sensed quantities of Section 2.3.1 are related to the elastic response of the phantom by a physical model, which plays the role of the measurement model of the sensing system: it predicts the instrumented outputs (probe–phantom contact force and surface-marker displacements) from the probe pose and a set of effective elastic and contact-model calibration parameters. Because the model-equivalent parameter set is not directly observable, it is inferred by evaluating this model in the inverse direction (Section 2.3.3). The model is implemented on the SOFA v23.06 platform [2] as a repeatable finite-element contact model of the probe–phantom interaction.

##### Forward Contact Model and Constraint Formulation

Each soft phantom is modeled as a deformable continuum discretized by a tetrahedral finite-element mesh. In the following formulation, bold uppercase letters denote matrices, bold lowercase or Greek letters denote vectors, and nonbold symbols denote scalars. The bold uppercase symbols F and L used for the measured and simulated quantities are exceptions: they denote three-dimensional contact-force and marker-displacement vectors, respectively. Let q, v, and a denote the nodal position, velocity, and acceleration vectors, respectively. The nodal position vector q is distinct from the parameter vector $x={\left[E,\mu ,d,D\right]}^{⊤}$ used for inverse identification. The semi-discrete equation of motion is

(1) $$Ma=f_{\mathrm{int}}\left(q;\;E,\mu \right)+f_{\mathrm{ext}}\left(t\right)+f_{c},$$

where M is the mass matrix, $f_{\mathrm{int}}$ is the internal elastic force parameterized by the effective elastic parameters (Young’s modulus *E* and Poisson’s ratio μ), $f_{\mathrm{ext}}$ includes external loads such as gravity, and $f_{c}$ denotes the contact reaction force induced by the probe–phantom interaction. Let $n_{q}$ denote the number of nodal translational degrees of freedom of the phantom. Then $M\in R^{n_{q}\times n_{q}}$, while q, v, a, $f_{\mathrm{int}}$, $f_{\mathrm{ext}}$, and $f_{c}$ belong to $R^{n_{q}}$. The implementation uses SOFA’s Tetrahedral Corotational FEM Force Field with method=large, i.e., a large-deformation corotational formulation with an isotropic linear elastic constitutive law. This retains only *E* and μ as material-related unknowns while updating element rotations during large deformation, keeping the inverse problem low-dimensional and computationally tractable under sparse multimodal measurements. Accordingly, *E* and μ are interpreted as model-equivalent parameters of the adopted FEM-contact model, rather than as complete constitutive properties of real soft tissue.

To enforce non-penetration, we adopt a constraint-based contact formulation following the Signorini contact law [1]. Let g(q) denote the normal gap function between the probe and the deformable surface (positive when separated and zero at contact). The unilateral contact condition is expressed as the complementarity system

(2) $$g\left(q\right)\geq 0,\;\delta \geq 0,\;\delta^{⊤}g\left(q\right)=0,$$

where δ is the vector of Lagrange multipliers associated with the contact constraints. For $n_{c}$ current contact constraints, $g\left(q\right),\delta \in R^{n_{c}}$. The constraint Jacobian $H\in R^{n_{c}\times n_{q}}$ therefore maps nodal variations to gap variations, and $H^{⊤}\delta \in R^{n_{q}}$ has the dimension of a nodal force vector. Linearizing the constraints yields the constraint Jacobian H=∂g/∂q, and the resulting contact reaction enters the semi-discrete dynamics as

(3) $$Ma=f_{\mathrm{int}}\left(q;\;E,\mu \right)+f_{\mathrm{ext}}\left(t\right)+H^{⊤}\delta .$$

Time discretization is performed using the Backward Euler scheme. At each time step, potential contacts are detected, and the complementarity constraints are resolved iteratively (Gauss–Seidel) to obtain contact-consistent state updates.

The model-predicted contact force is obtained from the resolved contact reaction term $f_{c}=H^{⊤}\delta$ and is reported as a 3D resultant force in the robot base frame, consistent with the F/T sensor measurements. In addition, the model tracks the displacements of the surface-marker nodes corresponding to the experimentally observed markers, enabling a time-synchronized comparison between measured and predicted deformation responses.

##### Forward Model Implementation and Outputs

Algorithm 1 summarizes the finite-element contact simulation used as the forward measurement model. The phantom is instantiated as a deformable tetrahedral mesh with parameterized elasticity and boundary conditions, while the probe is modeled as a rigid body whose pose is driven by the recorded robot end-effector trajectory. The model combines collision detection with the corotational large-deformation FEM formulation and an iterative constraint-based contact solver to enforce non-penetration and compute the deformation response; contact-model calibration parameters such as collision-detection thresholds are treated as configurable parameters. For indentation-based identification, the geometry reconstructed in the supine posture under physical gravity is used as the initial configuration. Simulation gravity is set to $g_{\mathrm{grav}}=0$, and only the incremental deformation induced by probe indentation is simulated. This initialization avoids applying an additional gravity-induced deformation to the already gravity-deformed reconstructed shape.

**Algorithm 1:** SOFA-Based Simulation of Probe–Breast Contact 1: **Procedure RootNode()** 2: RequiredPlugins▹ Collision detection, solvers, visualization 3: FreeMotionAnimationLoop 4: VisualStyle & gravity▹ Appearance; $g_{\mathrm{grav}}=0$ 5: CollisionPipeline: alarmDistance =d, contactDistance =D 6: GenericConstraintSolver (maxIterations =1000, tolerance $=10^{-6}$) 7: BreastNode: mesh, elasticity parameters (E,μ), fixed constraints 8: ProbeNode: mesh, initial pose 9: SimulationNode()   – Probe–breast: FEM, visual, collisionModel    ∘ rigidify()▹ Rigid probe    ∘ deformablePart: soft breast▹ Msh    ∘ rigidPart: probe▹ STL   – Update probe pose   – Collision detection   – SolversForDeformation: LinearSolverConstraintCorrection,    OdeSolver, SparseLDLSolver▹ Compute deformation   – Constraint solving and contact force computation   – Update marker positions  10: End simulation

Given a probe pose sequence and a candidate parameter set, the forward model outputs time-synchronized predictions of the resultant 3D probe–phantom contact force in the robot base frame and the 3D displacements of the surface-marker nodes corresponding to the experimentally tracked markers. These model-predicted outputs are directly comparable with the instrumented force and marker-displacement measurements and define the discrepancy that is minimized during inverse evaluation.

#### 2.3.3. Inverse Evaluation of the Measurement Model

Given the measurement model of Section 2.3.2, the model parameter set is evaluated by minimizing the discrepancy between the sensed and the model-predicted outputs. Each evaluation of the measurement model requires a finite-element contact simulation, so the number of admissible model evaluations is limited, and the inverse evaluation is therefore carried out with a surrogate-assisted numerical solver rather than by exhaustive search. The two sensed quantities are retained as two separate objectives, so that the solver is not free to trade one modality against the other implicitly; the consequence of this choice for the evaluated parameter set is examined by the single-modal ablation of Section 3.2.4.

The evaluated model parameter set comprises the model-equivalent effective elastic parameters *E* and μ and the contact-model calibration parameters *d* and *D*. Here, *E* and μ are parameters of the adopted isotropic linear FEM-contact model, inferred to reproduce the measured phantom response; they are not direct or absolute measurements of intrinsic breast-tissue material constants. They are collected in the parameter vector

(4) $$x={\left[E,\mu ,d,D\right]}^{⊤}.$$

Here, *d* denotes the alarm distance used to detect potential contact pairs before actual contact is enforced, while *D* denotes the contact distance threshold used to activate contact constraints. These two quantities mainly affect contact-onset timing, constraint generation, local force distribution, and numerical stability in the SOFA contact model. Therefore, *d* and *D* should be interpreted as contact-model calibration parameters rather than intrinsic biomechanical properties of the breast phantom. Including these contact-model calibration parameters in the inverse evaluation lets collision sensitivity and numerical stability adjust automatically, avoiding empirical manual tuning. To quantify the agreement between simulated outputs and experimental measurements, two normalized mean Euclidean-norm residual metrics are defined as

(5) $$\begin{array}{cc} f_{1}\left(x\right) & =\frac{1}{N}\sum_{i=1}^{N}\frac{{∥F_{i}^{\mathrm{sim}}\left(x\right)-F_{i}^{\exp}∥}_{2}}{\max_{1\leq k\leq N}{∥F_{k}^{\exp}-F_{0}^{\exp}∥}_{2}},\\ f_{2}\left(x\right) & =\frac{1}{M}\sum_{j=1}^{M}\frac{{∥L_{j}^{\mathrm{sim}}\left(x\right)-L_{j}^{\exp}∥}_{2}}{\max_{1\leq k\leq M}{∥L_{k}^{\exp}-L_{0}^{\exp}∥}_{2}}. \end{array}$$

where $F_{i}^{\mathrm{sim}},F_{i}^{\exp}\in R^{3}$ denote the simulated and measured contact-force vectors at the *i*th synchronized force sample, and $L_{j}^{\mathrm{sim}},L_{j}^{\exp}\in R^{3}$ denote the simulated and measured displacement vectors at the *j*th matched marker-displacement sample. The vectors $F_{0}^{\exp}$ and $L_{0}^{\exp}$ are the corresponding initial references; *N* and *M* are the numbers of valid force and displacement samples, respectively; and ${∥\cdot ∥}_{2}$ denotes the Euclidean norm. Thus, $f_{1}\left(x\right)$ and $f_{2}\left(x\right)$ are scalar normalized mean Euclidean-norm residuals.

Because each objective evaluation requires an expensive finite-element solution, the inverse problem is solved using the established ParEGO multiobjective Bayesian-optimization framework [19,35]. At each iteration, a random nonnegative weight vector is sampled,

(6) $$\lambda ={\left[\lambda_{1},\lambda_{2}\right]}^{⊤},\;\lambda_{1}+\lambda_{2}=1,\;\lambda_{j}\geq 0\;\left(j=1,2\right),$$

and the two normalized mean Euclidean-norm residuals are scalarized with a weighted Chebyshev function,

(7) $$S\left(x;\lambda ,z^{*}\right)=\max_{j=1,2}\lambda_{j}\left|f_{j}\left(x\right)-z_{j}^{*}\right|,$$

where $z^{*}$ is the ideal point formed by the best observed value of each objective. Thus, $\lambda ,z^{*}\in R^{2}$ are vectors, whereas $\lambda_{j}$, $z_{j}^{*}$, $f_{j}\left(x\right)$, and $S(x;\lambda ,z^{*})$ are scalars. This scalarization preserves an explicit trade-off between force consistency and deformation consistency instead of allowing one modality to dominate because of its physical units.

The scalar objective is modeled by Gaussian-process regression,

(8) $$S\left(x\right)∼\mathrm{GP}\left(m\left(x\right),k\left(x,x^{\prime }\right)\right),$$

where m(x) is the mean function and $k(x,x^{\prime })$ is a Matérn-5/2 kernel with a noise term. Hyperparameters are estimated by maximum likelihood. The next candidate is selected by maximizing the expected improvement,

(9) $$\mathrm{EI}\left(x\right)=E\left[\max\left(0,S_{\mathrm{best}}-S\left(x\right)\right)\right],$$

where $S_{\mathrm{best}}$ is the best scalarized value observed so far. All evaluated candidates are also ranked by nondominated sorting, and the nondominated set is retained as an approximate Pareto front. The representative parameter set used below is the point nearest the normalized ideal point.

The solver is a numerical tool and does not define the model-equivalent parameter set. Particle swarm optimization (PSO) and Gauss–Newton least squares (LS) are included only as computational baselines under the same measurement model.

The evaluation budget of the Bayesian solver consists of 30 initial samples drawn by Latin hypercube sampling followed by 25 sequential iterations, giving 55 evaluations of the measurement model per run.

## 3. Results

### 3.1. Experimental Setup and Evaluation Protocol

#### 3.1.1. Phantom Characterization

Three soft-tissue phantoms exhibiting distinct geometric and mechanical properties were employed to evaluate consistency across configurations, as shown in Figure 1. Phantoms A and B were custom-fabricated using poly(vinyl alcohol) (PVA) hydrogel with reagent ratios selected to mimic different stiffness levels [36]; Phantom A used 2 g PVA, 5 mL water, and 12.7 mL DMSO, whereas Phantom B used the same solvent composition with 5 g PVA. Phantom C was a commercial silicone-based standard (LYD810, Liuyedao Medical Technology, Ningbo, China) with complex surface curvature, providing a geometric-adaptability case [37]. All phantoms were instrumented with eight passive markers (red circular fiducials) to provide reference points for surface-deformation measurement.

#### 3.1.2. Data Acquisition Protocol

For each phantom, indentation tests were performed at five predefined locations with a maximum indentation depth of 10 mm. Measurements from the first four locations were used for inverse evaluation and the solver and modality comparisons; the fifth was excluded from all objectives and reserved for held-out validation. A robotic manipulator (UR5) equipped with a force/torque sensor (sampling at 1 kHz) and an eye-in-hand RGB-D camera executed controlled indentations. The force data and optical marker displacements were time-synchronized. This setup constitutes the physical measurement system and provides the force and displacement observations $F^{\exp}$ and $L^{\exp}$ defined in the method section, as shown in Figure 1.

#### 3.1.3. Alternative Inverse Solvers

The Bayesian solver used for inverse evaluation, denoted MBO in what follows, was compared against two standard alternatives so that the contribution of the solver could be separated from that of the sensing configuration. Particle swarm optimization (PSO) [22] served as the global optimization baseline, configured with a population size of 20 and a maximum of 15 iterations. In contrast, the least squares (LS) method, implemented via the Gauss–Newton algorithm with LS convergence tolerance, was employed as a representative gradient-based local optimizer. The search space for the model parameters was constrained to physically plausible ranges: E∈[3,30] kPa, μ∈[0.4,0.499], and contact-model calibration thresholds d,D∈[1,4] mm [38].

The three solvers do not consume the same number of measurement-model evaluations, and the evaluation count provides a hardware-independent cost measure because each evaluation is one finite-element contact solve. PSO uses a population of 20 over 15 iterations, i.e., 300 evaluations per run. The Bayesian solver uses 30 initial Latin-hypercube samples followed by 25 sequential iterations, i.e., 55 evaluations per run. Gauss–Newton LS terminates on its own convergence criterion. For the timing comparison, all three solvers were executed on the same laptop specified in Section 2.1 and used the same Python/SOFA forward-model implementation, reconstructed phantom model, measurements from the four identification locations, and parameter bounds. The reported wall-clock time covers a complete parameter-evaluation run, including optimizer overhead and all finite-element model evaluations.

To assess run-to-run variability without collecting additional measurements, the three inverse solvers were each executed ten times on Phantom A using the same force and marker-displacement observations, reconstructed geometry, parameter bounds, forward-model implementation, hardware, and solver-specific evaluation settings. Independent random seeds were used for PSO and MBO, while Gauss–Newton LS was initialized from ten independently generated parameter estimates. For each solver, the final force and displacement residuals and total wall-clock time were summarized as the mean and sample standard deviation across the ten runs.

### 3.2. Effect of the Inverse Solver on Residual Balance

To quantify how closely the measurement model reproduces the sensed quantities at the evaluated model parameter set, the normalized mean Euclidean-norm residuals for contact force ($\epsilon_{f}$) and surface deformation ($\epsilon_{l}$) were computed using the metrics defined in Equation (5). Table 1 compares the three inverse solvers (PSO, LS, MBO) across the three phantoms.

#### 3.2.1. Consistency of the Evaluated Model Parameter Set

The results show that the choice of solver affects the residual balance between force and surface-deformation prediction under the current phantom-based protocol, while the sensed quantities and the measurement model remain unchanged.

- Consistency Across Phantom Stiffness (Phantom A vs. B): Both PSO and MBO adapted to the stiffness variation, but MBO provided a more consistent balance between modalities. For the softer Phantom A, all methods achieved force residuals below 8%. For the stiffer Phantom B, MBO achieved the lowest joint residuals ($\epsilon_{f}=6.00\%$, $\epsilon_{l}=7.18\%$), whereas LS retained a higher deformation residual ($\epsilon_{l}=12.02\%$).
- Adaptability to Geometric Complexity: Phantom C, characterized by irregular surface curvature, posed a challenging evaluation case. LS produced the largest force residual among all trials ($\epsilon_{f}=17.67\%$). PSO achieved force and displacement residuals of $\epsilon_{f}=6.52\%$ and $\epsilon_{l}=8.23\%$, respectively, whereas MBO achieved a lower force residual ($\epsilon_{f}=4.93\%$) with a comparable displacement residual ($\epsilon_{l}=8.32\%$).

The three phantoms provide a limited but deliberately varied test range. Under MBO, the evaluated model-equivalent Young’s modulus ranged from 15.50 to 29.62 kPa, a 1.91-fold span, while the specimens included two PVA formulations with different stiffnesses and a silicone phantom with irregular surface curvature. Across this tested range, the force residual remained between 4.93% and 6.00%, and the displacement residual between 7.18% and 12.33%. These results indicate that the method can evaluate model-equivalent parameter sets for the tested changes in stiffness, material type, and geometry. Because the geometry was reconstructed and a parameter set was evaluated separately for each phantom, however, this evidence concerns adaptability through re-identification rather than transfer of one calibrated parameter set across different objects.

#### 3.2.2. Cost of Evaluating the Model Parameter Set

Whether the model-equivalent parameter set can be evaluated at acceptable cost determines whether the sensing system is usable for offline calibration before a soft-tissue simulation or grasp-planning workflow. Table 1 reports the total wall-clock time for each solver, complementing the measurement-model evaluation counts described above. The evaluation count provides a hardware-independent measure of finite-element cost, whereas the recorded time gives the realized end-to-end computational cost on the common platform described above.

- Bayesian solver versus PSO: PSO used 300 evaluations per phantom, whereas the Bayesian solver used 55, a 5.5-fold reduction in finite-element evaluations. Across the three phantom-specific runs, the total wall-clock times were 4997.7 s for PSO and 377.1 s for MBO, corresponding to averages of 1665.9 and 125.7 s, respectively. Thus, the observed average MBO time was 92.5% lower than that of PSO (a 13.3-fold speedup on this platform) while retaining comparable or lower residuals across the tested phantoms.
- Bayesian solver versus LS: The LS runs totaled 690.8 s, with an average of 230.3 s; the corresponding average MBO time was 45.4% lower. LS was slightly faster only for Phantom C (60.1 versus 67.0 s), but its force residual there was 17.67%, the largest of any trial. MBO therefore provided the most favorable observed balance between total computation time and residual consistency under the common experimental implementation.

Table 2 summarizes the repeated-run results on Phantom A. All runs returned an evaluated parameter set. MBO produced force and displacement residuals of 5.56±0.51% and 12.55±0.34%, respectively. Compared with PSO, its mean force residual was slightly higher, and its mean displacement residual was slightly lower, while the standard deviations of both residuals were smaller. Its mean wall-clock time was 112.3 s, compared with 1672.3 s for PSO and 190.2 s for LS. LS produced higher mean residuals and greater dispersion than MBO. These results indicate consistent objective-level performance and computational cost for MBO under the Phantom A configuration; the repeated-run analysis does not establish identical variability for the other phantom configurations.

#### 3.2.3. Predictive Validation on Held-Out Data

To assess whether the evaluated model-equivalent parameters maintain predictive consistency beyond the locations used for optimization, the parameters obtained from the first four indentation locations were directly applied to the fifth held-out location. This fifth location was excluded from the parameter evaluation process and was used only for predictive validation. For each solver, the complete evaluated vector [E,μ,d,D] was transferred without re-estimation, so the following force and deformation results assess the corresponding configured FEM-contact model rather than the elastic parameters alone.

Force Response Analysis: Figure 4 compares the measured force-depth curves against model predictions at this held-out location. The experimental data exhibited distinct nonlinear force–depth characteristics under indentation.

- For Phantoms B and C, the MBO-predicted responses demonstrated high consistency with the measured force–depth curves.
- In contrast, the LS prediction for Phantom C showed increasing deviation as the indentation depth increased beyond 6 mm, underestimating the contact force. This divergence at large deformations indicates that the model-equivalent parameters evaluated by LS failed to capture the force response under larger indentation within the adopted linear-elastic contact model.
- For Phantom B, MBO maintained the tightest fit, whereas PSO showed a slight underestimate in the deep-indentation region, consistent with the residual balance observed for MBO under the current setup.

Deformation Tracking Analysis: Figure 5 presents the marker-wise displacement comparison for Phantom A. This analysis reveals the spatial distribution of the prediction error:

- Dominant Deformation Axes: For markers experiencing significant displacement (e.g., *X*-axis), the MBO predictions closely tracked the physical measurements. This indicates that the FEM-contact model configured by the transferred [E,μ,d,D] set reproduces the dominant deformation trend at the held-out location.
- Measurement-Uncertainty Effect: For markers undergoing submillimeter motion, measurement uncertainty may contribute appreciably to the observed discrepancies. These residuals reflect both measurement uncertainty and model discrepancy, whose individual contributions cannot be separated from the present data.

Overall, the held-out results support predictive consistency of the complete FEM-contact model configured by the MBO-evaluated parameter set under the adopted phantom protocol.

#### 3.2.4. Contribution of Force–Vision Fusion

The inverse evaluation was repeated with each sensed quantity used alone to test whether both channels are necessary. Table 3 shows that optimizing one channel improves its own residual but can degrade prediction of the unobserved response. With PSO, the force-only objective reduced the force residual to 3.63% on Phantom C, whereas its displacement residuals reached 15.10% on Phantom A. Conversely, the displacement-only objective reduced the displacement residual to 7.16% on Phantom C but increased its force residual to 20.11%. The same cross-channel imbalance is more pronounced in several LS results. Neither single-modality objective therefore provides consistent agreement with both sensed quantities across all three phantoms.

This complementarity is consistent with the displacement-controlled protocol: *E* primarily scales the reaction force, whereas μ affects the deformation pattern. Contact force and surface displacement consequently constrain different aspects of the configured model. These results therefore support joint force–vision sensing for balanced force–deformation reproduction under the present protocol.

### 3.3. FEM-Contact Analysis Under Posture Changes

To provide a preliminary evaluation of whether the calibrated measurement model remains useful under posture changes, a FEM-contact posture-transition experiment was conducted. A new breast phantom was fabricated using the same material ratio as Phantom A, with a small amount of red ink added to improve visual observation. This posture phantom was calibrated independently from its own indentation measurements; the numerical parameter set reported for Phantom A was not assigned to it on the basis of the shared formulation. The common material ratio was used to control material variation so that the posture experiment could focus on the change in loading orientation. Indentation measurements were first used to evaluate the complete model parameter vector $x^{*}={\left[E^{*},\mu^{*},d^{*},D^{*}\right]}^{⊤}$. The elastic parameters $E^{*}$ and $\mu^{*}$ define the model-equivalent constitutive response within the adopted FEM representation, whereas $d^{*}$ and $D^{*}$ retain the calibrated collision-detection and contact-constraint settings of the same SOFA model. All four values were carried forward within this phantom’s model and held fixed; no parameter was re-estimated from the posture data. Accordingly, this protocol evaluates within-phantom transfer from indentation calibration to posture loading, rather than transfer of a fixed numerical parameter set between phantoms or materials.

The phantom, mounted on a rigid torso mannequin, was first placed supine and then physically rotated to four lateral postures. For each lateral posture, surface observations were captured from two camera perspectives. The relative transformation between the supine and lateral postures was obtained using the AprilTag/robot coordinate system [39]. In simulation, the calibrated FEM-contact model, including $x^{*}$, the mesh topology, and the fixed boundary constraints of the supine model, was preserved. The posture transition was represented by changing the gravity direction in the local model frame, after which the quasi-static equilibrium configuration was solved. Thus, gravity supplies the posture-dependent load, while $E^{*}$, $\mu^{*}$, $d^{*}$, and $D^{*}$ retain the material-response and contact-model configuration established during indentation. The initial state denotes rigid posture transformation without deformation compensation, whereas the FEM-contact result denotes the predicted gravity-driven and probe compression deformation. Performance was quantified by the surface-fitting RMSE between the observed point cloud and model surface and by the marker-position RMSE between predicted and measured lateral-posture marker positions.

As shown in Figure 6, the calibrated FEM-contact prediction improved both surface fitting and marker-position consistency compared with the initial state. The mean surface fitting error decreased from 2.86 mm to 2.18 mm, corresponding to a reduction of 23.76%. Meanwhile, the mean marker-position error decreased from 5.48 mm to 2.36 mm, corresponding to a reduction of 56.94%. These results indicate that the complete model configured by $x^{*}$ reproduced the dominant deformation tendency during controlled supine-to-lateral posture changes. The improved marker correspondence further suggests that the predicted deformation field could provide a useful prior for transferring surface keypoints across postures.

## 4. Discussion

### 4.1. Model-Equivalent Parameters and Multimodal Constraints

This study developed a force–vision sensing system and a finite-element contact measurement model for indirect evaluation of soft-phantom mechanical response. The single-modality results in Table 3 clarify the role of sensor fusion: force principally constrains stiffness under displacement-controlled indentation, whereas surface motion supplies deformation-shape information. The deterioration of the omitted channel under either single-modality objective is observed across phantoms and solvers, supporting the use of both channels as a property of the sensing configuration rather than a consequence of one optimizer.

The reported quantities should be interpreted at the level at which they are evaluated. The elastic parameters *E* and μ are model-equivalent effective parameters of the adopted isotropic linear FEM representation; they are not direct or absolute measurements of intrinsic material constants or a complete constitutive description of heterogeneous soft tissue. Likewise, *d* and *D* are contact-model calibration parameters. Accordingly, the output is a calibrated parameter set for reproducing the measured probe–phantom interaction within the stated model and protocol, rather than a direct measurement of intrinsic tissue properties.

A nonlinear force–depth response does not necessarily imply a nonlinear local constitutive law. In the present forward model, element rotations, evolving deformed geometry, changing probe–phantom contact area, and activation of unilateral contact constraints can produce a nonlinear global indentation response even though the local elastic constitutive law is linear. These effects can contribute to the curvature reproduced by the fitted model over the calibrated 0–10 mm indentation range; their individual contributions were not separately quantified. However, the effects of unmodeled hyperelasticity, viscoelasticity, or heterogeneity may be partially compensated for by adjustments to the fitted elastic parameters *E* and μ and contact parameters *d* and *D*, or they may remain as residual discrepancies between predictions and measurements. Accordingly, *E* and μ are loading-range- and model-dependent effective quantities rather than intrinsic constants. Low residuals within the calibration and held-out protocol do not establish constitutive validity, and extrapolation to deeper indentation or different loading paths may introduce systematic bias.

Two independent uses of data not included in the inverse objective provide complementary checks on this calibrated model. At the fifth indentation location, the complete transferred parameter set predicts force and marker displacement without re-estimation. In the posture experiment, the same four-parameter configuration is held fixed while only the gravity direction is changed, reducing both surface-fitting and marker-position errors. These results test transfer beyond the calibration locations and loading orientation. Because the complete vector is held fixed within each corresponding phantom model, the improvements provide evidence for the predictive consistency of the configured FEM-contact model as a whole rather than for any parameter in isolation.

This validity should not be extrapolated to phantoms with substantially different properties. Changes in stiffness, geometry, constitutive behavior, or support conditions can alter the model-equivalent parameter set. For a new phantom, the geometry should therefore be reconstructed and *E* and μ re-evaluated; *d* and *D* may also require recalibration if the mesh or contact conditions change. The three-phantom results demonstrate application of the same identification workflow through object-specific re-identification, not direct transfer of one numerical parameter set across materials.

### 4.2. Relevance to Soft-Tissue sim2real Transfer

The present implementation is intended for controlled phantom evaluation and offline calibration. Within that scope, three geometrically and mechanically distinct phantoms, multimodal residuals, a held-out indentation location, and controlled posture changes, provide evidence for sensing complementarity and predictive consistency. PSO and LS are included only as computational baselines under the same observations and measurement model; the comparison is not a claim about alternative biomechanical characterization instruments. Extension to biological tissue will require the model class and sensing protocol to be adapted to the corresponding anatomy, heterogeneity, and loading conditions.

For sim2real use, the potential value of the identified model-equivalent parameters lies in improving the physical fidelity of the simulated soft tissue. By calibrating the finite-element model against measured force and deformation responses, the simulator may reproduce the measured contact behavior more consistently than a model based only on nominal or arbitrarily assigned parameters. This calibration can reduce one source of mismatch between simulated and real contact mechanics and may help narrow the sim-to-real gap. A more representative soft-tissue model may, in turn, provide a useful environment for developing, training, and evaluating force-aware grasping and contact-rich manipulation policies before subsequent deployment to real robotic systems.

### 4.3. Human-Inspired Active Perception for Future Grasping

The calibrated parameter set defines a useful interface to future robotic grasping. Humans assess the compliance of an unfamiliar object primarily through tactile and force feedback during exploratory touch, without requiring prior knowledge of its intrinsic Young’s modulus or even direct visual observation. Vision nevertheless provides complementary information about object geometry, deformation, and motion and becomes particularly valuable when manipulating highly deformable or mobile soft objects whose shape changes substantially during interaction. The proposed framework can therefore be viewed as a computational analog of this tactile-dominant multisensory process: a robot performs a controlled exploratory contact, measures the contact force together with visible deformation, evaluates a model-equivalent parameter set, and then uses the calibrated model for action selection.

A gripper equipped with force and vision sensing could use this cycle to estimate the effective compliance of soft objects and select an appropriate contact trajectory, preload, or grasp configuration. In this process, force feedback provides the primary cue for mechanical interaction, while vision complements it by observing object shape and deformation, especially during large-deformation manipulation. The parameterized FEM model could then be queried to predict deformation and contact force before execution, forming a sense–identify–simulate–plan/control loop. These implications are prospective: no gripper integration, object-recognition benchmark, or grasp-control experiment is claimed here. Future work should evaluate online identification, uncertainty-aware model updating, and closed-loop grasp planning with anatomically realistic and heterogeneous phantoms.

### 4.4. Limitations

The study is limited to breast-shaped PVA and silicone phantoms, sparse surface markers, a linear isotropic constitutive approximation, and offline optimization. The present three-phantom data do not establish performance beyond the specific material formulations and geometries tested; systematic validation should vary stiffness, constitutive behavior, and geometry independently over a broader factorial test set. The corotational/contact formulation captures a nonlinear global indentation response, but the linear constitutive law does not represent hyperelasticity, viscoelasticity, or heterogeneity; consequently, the evaluated parameters may vary with the mesh, boundary conditions, contact formulation, loading range, and sensor uncertainty. The posture-change results support predictive consistency across loading orientations only for one independently calibrated phantom; they do not establish that a numerical parameter set transfers across different materials, objects, or manipulation tasks. Sparse marker observations and the absence of an independent uncertainty assessment of the complete measurement chain limit the interpretation of small-motion residuals. The identified *E* and μ characterize the quasi-static indentation response within the present model and should not be interpreted as frequency-dependent effective properties governing wave propagation or vibroacoustic transmission.

## 5. Conclusions

This study developed a force–vision multimodal framework that combines finite-element contact modeling with multiobjective Bayesian optimization to identify model-equivalent elastic and contact parameters from measured forces and surface deformation. Across three phantoms, MBO achieved normalized force and displacement residuals of 4.93%–6.00% and 7.18%–12.33%, respectively, using 55 model evaluations, compared with 300 evaluations for PSO. Single-modality trials showed that force and vision provide complementary constraints: optimizing either channel alone could reduce its own residual while degrading prediction of the unobserved response. At a held-out indentation location, the calibrated model provided consistent force and displacement predictions without parameter re-estimation under the corresponding phantom-specific setup. Under controlled posture changes, holding the posture phantom’s independently calibrated parameter set fixed while changing the gravity direction reduced the surface-fitting RMSE from 2.86 to 2.18 mm and the marker-position RMSE from 5.48 to 2.36 mm. These results support a sample-efficient approach to calibrating soft-object simulation models for reproducing contact force and deformation within the tested phantom protocol.

## Figures and Tables

**Figure 1:**
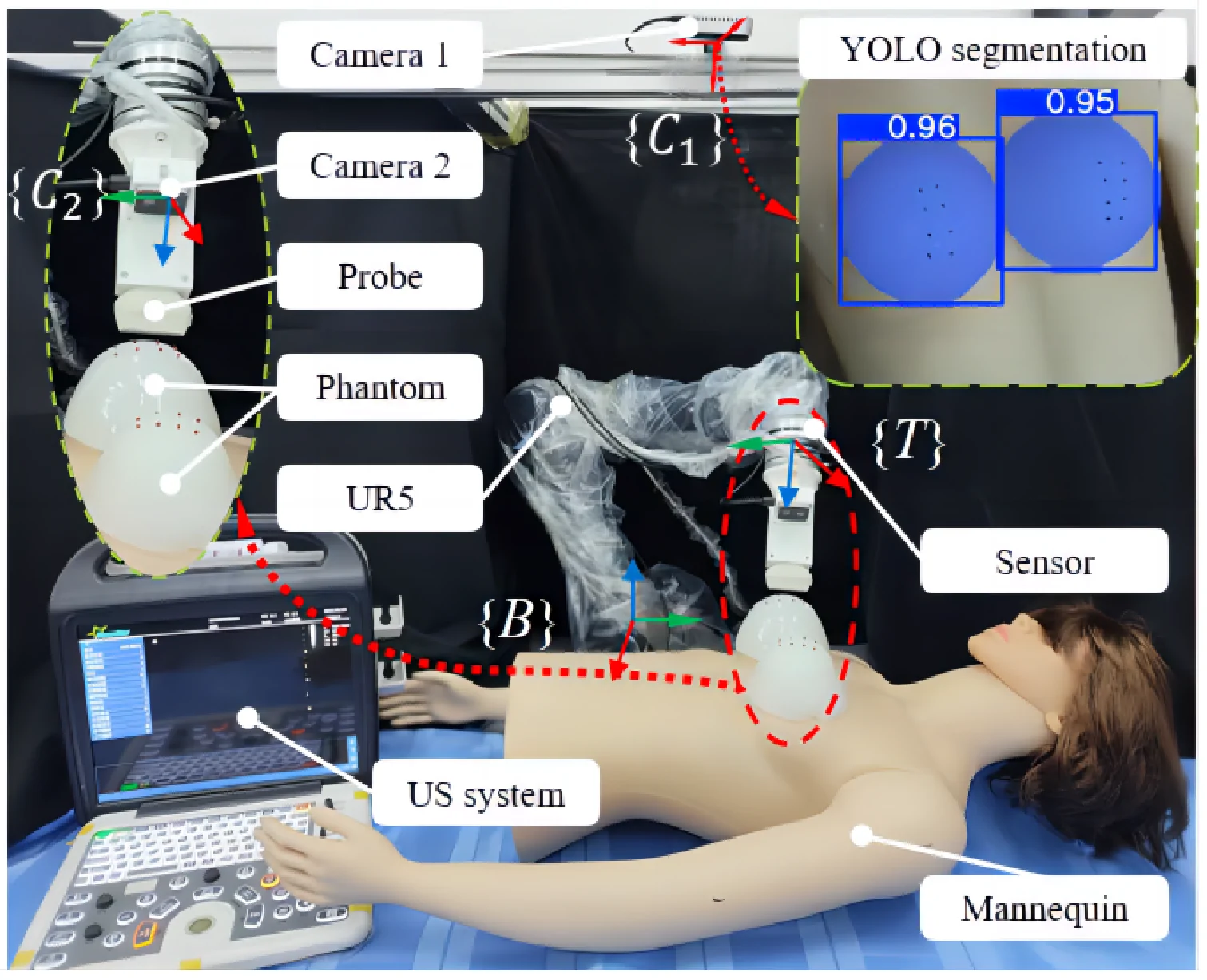
Force–vision measurement system used to collect contact force and surface deformation during controlled probe–phantom interaction.

**Figure 2:**
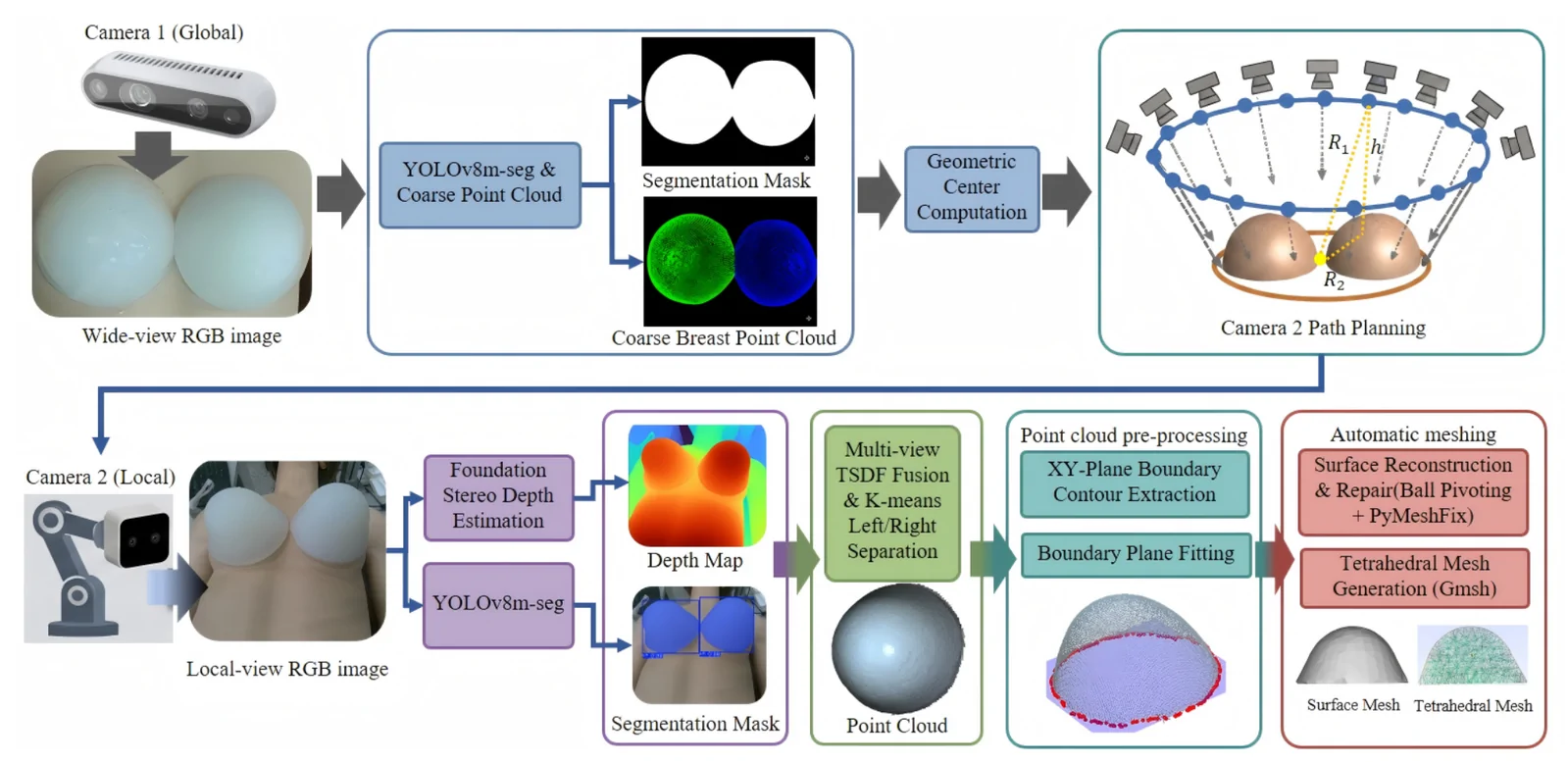
Overall workflow of model geometry acquisition, from multi-view RGB-D data acquisition to finite element mesh generation.

**Figure 3:**
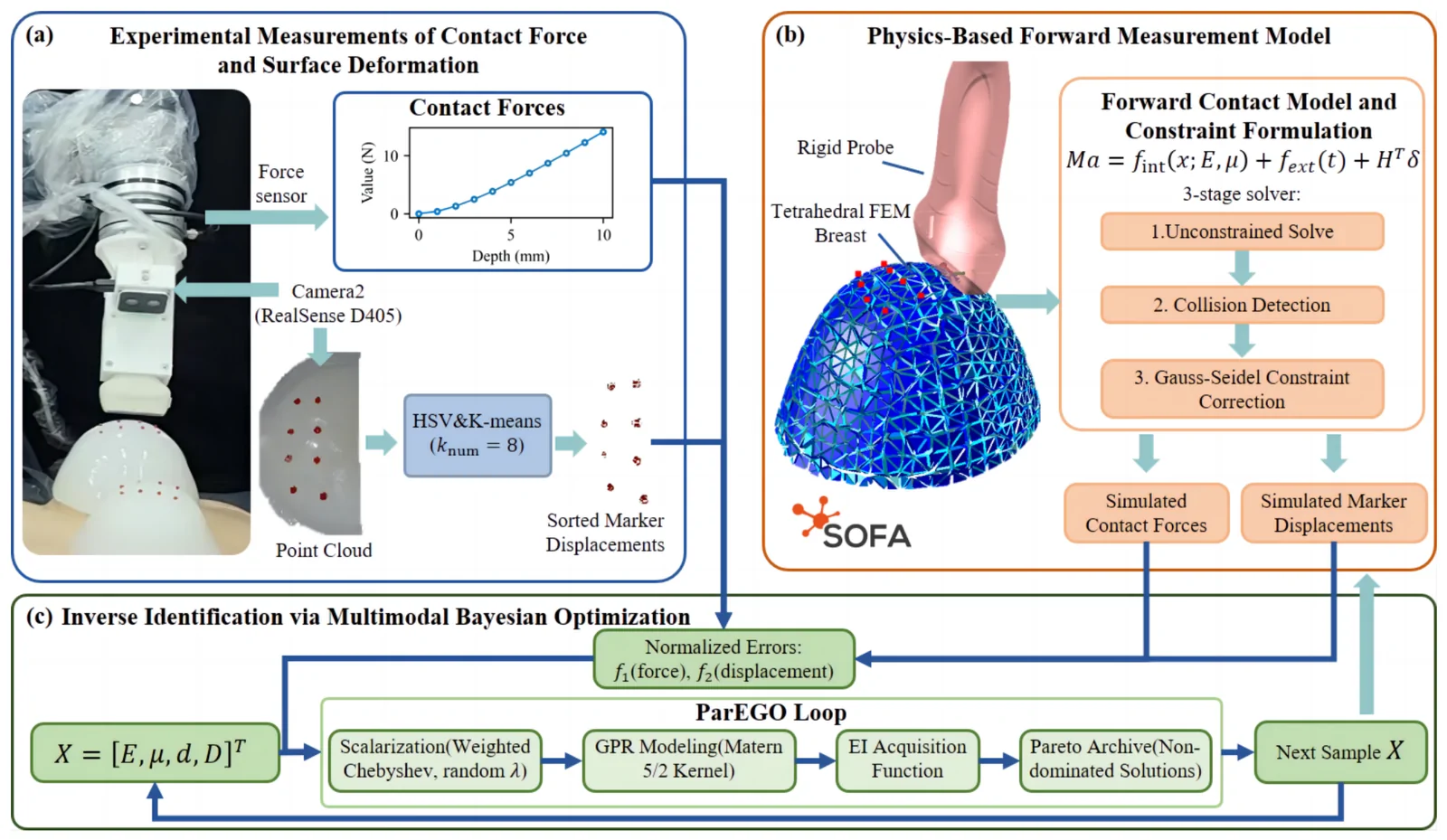
Sensed quantities and inverse evaluation: (**a**) contact force and surface deformation measured by the physical system; (**b**) the corresponding quantities predicted by the SOFA measurement model; and (**c**) inverse evaluation of the measurement model.

**Figure 4:**
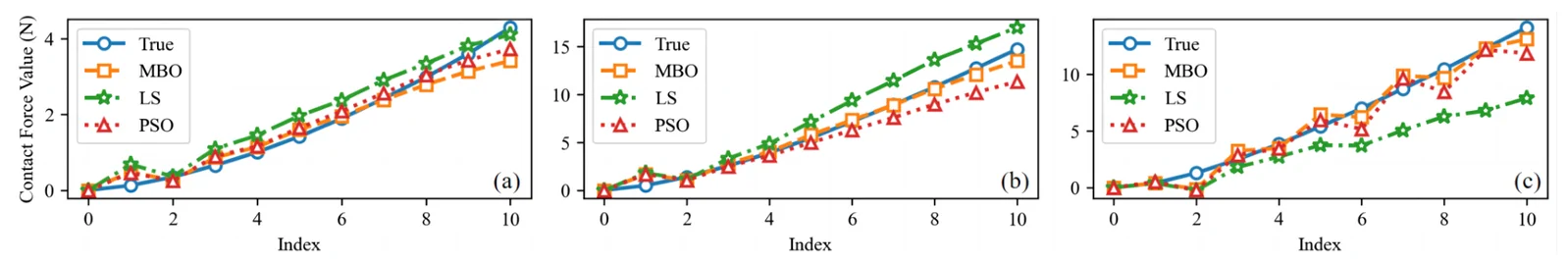
Held-out validation of contact force responses at the fifth indentation location using parameter sets evaluated by PSO, LS, and MBO for three soft-tissue phantoms (**a**–**c**). The fifth location was excluded from parameter evaluation, and the predicted forces were generated by the finite-element forward model using the first four locations.

**Figure 5:**
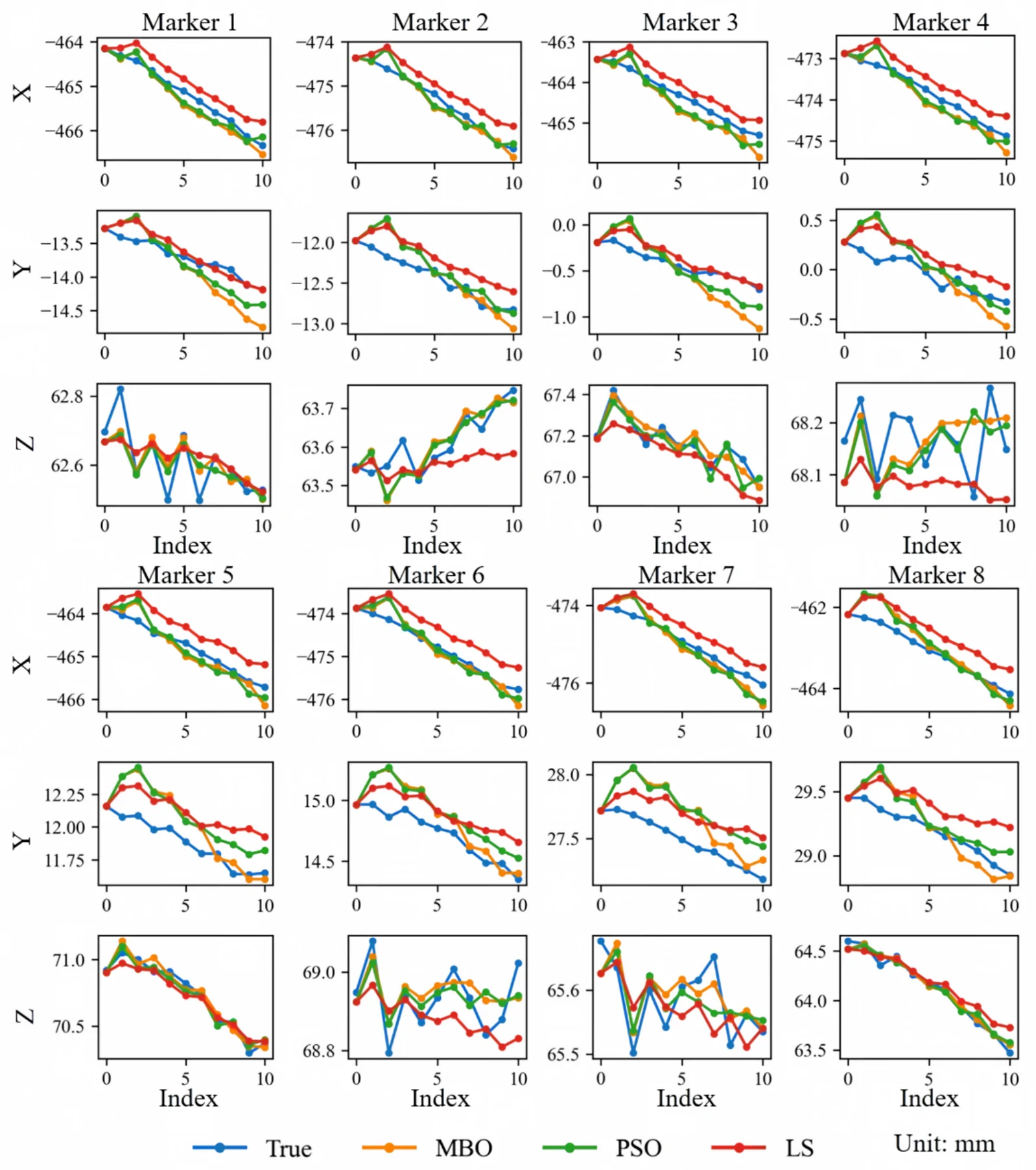
Held-out validation of surface deformation predictions for Phantom A at the fifth indentation location. Measured marker trajectories are compared with finite-element predictions using parameter sets evaluated by PSO, LS, and MBO from the first four indentation locations. The upper three rows show the *X*-, *Y*-, and *Z*-component trajectories of Markers 1–4; the lower three rows show the corresponding trajectories of Markers 5–8.

**Figure 6:**
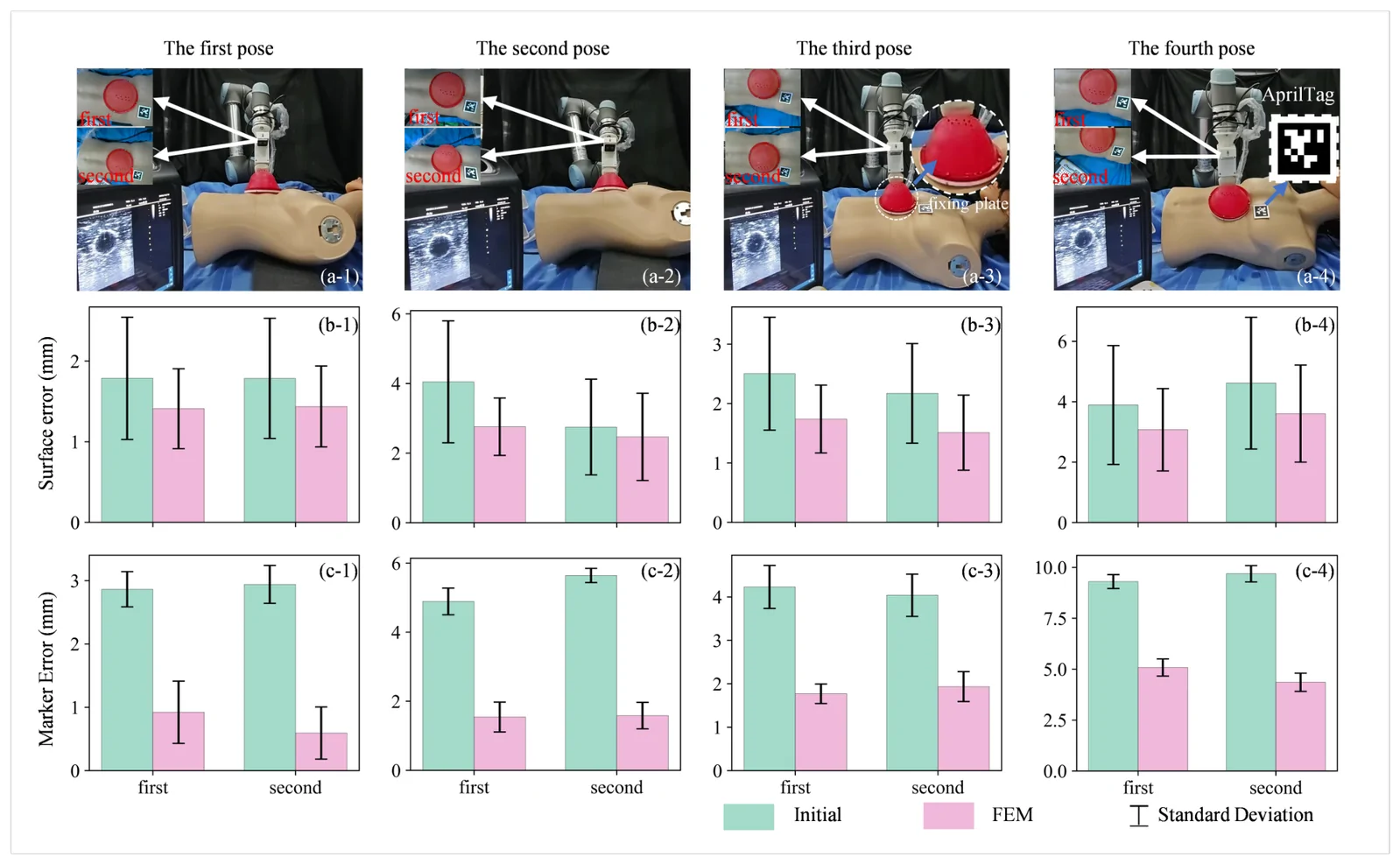
Posture-change validation using the calibrated FEM-contact model with the independently calibrated parameter set $\left[E^{*},\mu^{*},d^{*},D^{*}\right]$ held fixed during posture changes. (**a**) Single-view RGB images of four lateral postures; (**b**) surface-fitting errors before and after FEM-contact deformation compensation; (**c**) marker-position errors before and after compensation. All four parameters were evaluated from this phantom’s own indentation data and remained fixed during posture simulation.

**Table 1:** Model-equivalent parameter sets and total wall-clock times for the three inverse solvers under common measurements, forward-model implementation, and hardware.

Phantom	Method	*E* (kPa)	μ	*d* (mm)	*D* (mm)	$\epsilon_{f}$ (%)	$\epsilon_{l}$ (%)	Total Time (s)
A	PSO	14.60	0.450	2.07	1.17	4.90	12.95	1661.7
A	LS	16.92	0.443	2.19	1.41	7.58	13.78	187.6
A	MBO	15.50	0.401	2.13	1.25	5.95	12.33	110.9
B	PSO	28.95	0.437	3.38	2.59	6.98	9.84	2728.8
B	LS	28.68	0.490	2.87	2.19	10.06	12.02	443.1
B	MBO	29.62	0.473	2.60	2.37	6.00	7.18	199.2
C	PSO	23.00	0.490	3.45	2.47	6.52	8.23	607.2
C	LS	22.15	0.470	2.52	2.40	17.67	11.11	60.1
C	MBO	23.88	0.490	3.48	2.56	4.93	8.32	67.0

**Table 2:** Run-to-run variability of the three inverse solvers on Phantom A over ten runs. Values are reported as mean ± sample standard deviation.

Phantom	Solver	$\epsilon_{f}$ (%)	$\epsilon_{l}$ (%)	Total Time (s)
A	MBO	5.56±0.51	12.55±0.34	112.3±3.2
A	PSO	5.02±1.01	13.02±0.56	1672.3±60.5
A	LS	7.64±1.52	13.99±1.65	190.2±30.2

**Table 3:** Model-Equivalent Parameter Sets and Prediction Errors Evaluated Under Single-Modality Objectives.

Phantom	Method	*E* (kPa)	μ	*d* (mm)	*D* (mm)	$\epsilon_{f}$ (%)	$\epsilon_{l}$ (%)	Time (s)
A	Force–PSO	13.46	0.469	3.50	1.11	4.08	15.10	1579.3
A	Force–LS	15.50	0.445	2.50	1.17	5.49	14.16	226.8
B	Force–PSO	25.78	0.483	3.51	2.42	3.73	13.15	2789.2
B	Force–LS	22.47	0.465	2.50	3.15	9.29	15.24	87.3
C	Force–PSO	23.79	0.492	3.41	1.15	3.63	10.33	701.5
C	Force–LS	25.57	0.477	2.50	2.01	12.99	18.97	44.2
A	Disp.–PSO	18.23	0.464	2.17	1.01	6.56	12.31	1762.5
A	Disp.–LS	15.50	0.449	2.50	2.52	13.23	17.93	83.5
B	Disp.–PSO	24.19	0.479	3.34	1.91	10.15	8.16	2594.6
B	Disp.–LS	15.50	0.449	2.50	2.50	22.32	9.02	75.5
C	Disp.–PSO	21.02	0.413	3.71	3.17	20.11	7.16	606.2
C	Disp.–LS	15.50	0.449	2.50	2.81	32.16	16.71	27.6

## Data Availability

The original contributions presented in this study are included in the article material. Further inquiries can be directed to the corresponding author.

## References

[1] Duriez C., Andriot C., Kheddar A. (2004). Signorini’s contact model for deformable objects in haptic simulations. Proceedings of the 2004 IEEE/RSJ International Conference on Intelligent Robots and Systems (IROS).

[2] Faure F., Duriez C., Delingette H., Allard J., Gilles B., Marchesseau S., Talbot H., Courtecuisse H., Bousquet G., Peterlik I. (2012). Sofa: A multi-model framework for interactive physical simulation. Soft Tissue Biomechanical Modeling for Computer Assisted Surgery.

[3] Göbel B., Huurdeman J., Reiterer A., Möller K. (2025). Robot-Based Procedure for 3D Reconstruction of Abdominal Organs Using the Iterative Closest Point and Pose Graph Algorithms. J. Imaging.

[4] Chen T., Zhao X., Zheng G., Wang J., Ling Q., Tao B., Yin Z. (2024). Real-time deformable registration for robotic ultrasound surgical guidance based on vectorized trust-region optimization. IEEE Trans. Instrum. Meas..

[5] Lan S., Li X., Guo Z. (2023). DRT: Deformable region-based transformer for nonrigid medical image registration with a constraint of orientation. IEEE Trans. Instrum. Meas..

[6] Cao Z., Wang Y., Zhao H., Ding H., Zhang S. (2025). Robust Robotic Breast Ultrasound Scanning and Real-time Lesion Localization. Proceedings of the 2025 IEEE International Conference on Robotics and Automation (ICRA).

[7] Firouzi N. (2022). On skin growth using hyperelastic membrane model. J. Braz. Soc. Mech. Sci. Eng..

[8] Firouzi N., Amabili M. (2024). Two-dimensional growth of incompressible and compressible soft biological tissues. Eur. J. Mech.–A/Solids.

[9] Mazier A., Bordas S.P. (2024). Breast simulation pipeline: From medical imaging to patient-specific simulations. Clin. Biomech..

[10] Eiben B., Vavourakis V., Hipwell J.H., Kabus S., Lorenz C., Buelow T., Williams N.R., Keshtgar M., Hawkes D.J. (2016). Surface driven biomechanical breast image registration. Proceedings of the Medical Imaging 2016: Image-Guided Procedures, Robotic Interventions, and Modeling.

[11] Awwad H., García E., Martí R. (2024). Graph Neural Networks for modelling breast biomechanical compression. Proceedings of the Deep Breast Workshop on AI and Imaging for Diagnostic and Treatment Challenges in Breast Care.

[12] Chen J.H., Chan S., Zhang Y., Li S., Chang R.F., Su M.Y. (2019). Evaluation of breast stiffness measured by ultrasound and breast density measured by MRI using a prone-supine deformation model. Biomark. Res..

[13] Mîra A., Carton A.K., Muller S., Payan Y. (2018). A biomechanical breast model evaluated with respect to MRI data collected in three different positions. Clin. Biomech..

[14] Zarastvand M.R., Abdoli E., Talebitooti R. (2025). A lattice metamaterial-based sandwich cylindrical system for numerical simulation approach of vibroacoustic transmission considering triply periodic minimal surface. Appl. Math. Mech.-Engl. Ed..

[15] Fougeron N., Rohan P.Y., Haering D., Rose J.L., Bonnet X., Pillet H. (2020). Combining freehand ultrasound-based indentation and inverse finite element modeling for the identification of hyperelastic material properties of thigh soft tissues. J. Biomech. Eng..

[16] Chen B., Starman B., Halilovič M., Berglund L.A., Coppieters S. (2025). Finite element model updating for material model calibration: A review and guide to practice. Arch. Comput. Methods Eng..

[17] Oddes Z., Solav D. (2023). Identifiability of soft tissue constitutive parameters from in-vivo macro-indentation. J. Mech. Behav. Biomed. Mater..

[18] Hu Z., Liao S., Zhou J., Chen Q., Wu R. (2024). Elastic parameter identification of three-dimensional soft tissue based on deep neural network. J. Mech. Behav. Biomed. Mater..

[19] Borowska A., Gao H., Lazarus A., Husmeier D. (2022). Bayesian optimisation for efficient parameter inference in a cardiac mechanics model of the left ventricle. Int. J. Numer. Methods Biomed. Eng..

[20] Ross C.J., Laurence D.W., Aggarwal A., Hsu M.C., Mir A., Burkhart H.M., Lee C.H. (2024). Bayesian optimization-based inverse finite element analysis for atrioventricular heart valves. Ann. Biomed. Eng..

[21] Shahriari B., Swersky K., Wang Z., Adams R.P., De Freitas N. (2015). Taking the human out of the loop: A review of Bayesian optimization. Proc. IEEE.

[22] Wang D., Tan D., Liu L. (2018). Particle swarm optimization algorithm: An overview. Soft Comput..

[23] Fu E., Zhou H., de la Lastra Á.A., Zhang R., Ayyala H., Broyles J., Pomahac B., Jagadeesan J. (2024). SLAM-Based Breast Reconstruction System for Surgical Guidance Using a Low-Cost Camera. IEEE Trans. Med. Robot. Bionics.

[24] Wen B., Trepte M., Aribido J., Kautz J., Gallo O., Birchfield S. (2025). FoundationStereo: Zero-Shot Stereo Matching. Proceedings of the IEEE/CVF Conference on Computer Vision and Pattern Recognition (CVPR).

[25] von Haxthausen F., Böttger S., Wulff D., Hagenah J., García-Vázquez V., Ipsen S. (2021). Medical robotics for ultrasound imaging: Current systems and future trends. Curr. Robot. Rep..

[26] Li K., Xu Y., Meng M.Q.H. (2021). An overview of systems and techniques for autonomous robotic ultrasound acquisitions. IEEE Trans. Med. Robot. Bionics.

[27] Jiang Z., Salcudean S.E., Navab N. (2023). Robotic ultrasound imaging: State-of-the-art and future perspectives. Med. Image Anal..

[28] Tan J., Li J., Li Y., Li B., Leng Y., Rong Y., Fu C. (2024). Autonomous Trajectory Planning for Ultrasound-Guided Real-Time Tracking of Suspicious Breast Tumor Targets. IEEE Trans. Autom. Sci. Eng..

[29] Zhang L., Yang D., Niu B., Yang H., Huang Q., Jiang L., Liu H. (2025). Smooth Path Planning and Dynamic Contact Force Regulation for Robotic Ultrasound Scanning. IEEE Robot. Autom. Lett..

[30] Niu B., Yang D., Zhang L., Ji Y., Jiang L., Liu H. (2025). Enhancing ultrasound scanning skills in a leader–follower robotic system through expert hand impedance regulation. IEEE J. Biomed. Health Inform..

[31] Zhou Y., Jiang L., Wang P., Niu B., Liu H. (2026). Path Generation and Stable Interaction Control for Autonomous Robotic Breast Ultrasound Scanning. IEEE Trans. Autom. Sci. Eng..

[32] Niu B., Yang D., Zhang L., Ji Y., Geng T., Bian G. (2026). Human–Robot Shared Control Strategy for Ultrasound Scanning Based on Lesion Position Prediction. IEEE Trans. Autom. Sci. Eng..

[33] Hussain M. (2023). YOLO-v1 to YOLO-v8, the rise of YOLO and its complementary nature toward digital manufacturing and industrial defect detection. Machines.

[34] Curless B., Levoy M. A volumetric method for building complex models from range images. Proceedings of the 23rd Annual Conference on Computer Graphics and Interactive Techniques.

[35] Knowles J. (2006). ParEGO: A hybrid algorithm with on-line landscape approximation for expensive multiobjective optimization problems. IEEE Trans. Evol. Comput..

[36] Niu B., Yang D., Wang P., Yang H., Zhang L., Gu Y., Jiang L. (2023). Virtual-force-guided intraoperative ultrasound scanning with online lesion location prediction: A phantom study. Int. J. Med. Robot. Comput. Assist. Surg..

[37] Zhang L., Yang D., Yang H., Niu B., Huang Q., Liu H. (2025). Circular path planning and adaptive force control for breast ultrasound autonomous scanning. Int. J. Intell. Robot. Appl..

[38] Ramião N.G., Martins P.S., Rynkevic R., Fernandes A.A., Barroso M., Santos D.C. (2016). Biomechanical properties of breast tissue, a state-of-the-art review. Biomech. Model. Mechanobiol..

[39] Olson E. (2011). AprilTag: A robust and flexible visual fiducial system. Proceedings of the 2011 IEEE International Conference on Robotics and Automation.

